# Assessing sociology and psychology in UK undergraduate medical education: Square peg in a round hole?

**DOI:** 10.15694/mep.2021.000133.1

**Published:** 2021-05-17

**Authors:** Jeni Harden, Tracey Collett, Simon Forrest, Kathleen Kendall

**Affiliations:** 1 University of Edinburgh; 2 University of Plymouth; 3 University of Durham; 4 University of Southampton

**Keywords:** Sociology and Psychology, Assessment, Social and Behavioural Sciences

## Abstract

This article was migrated. The article was marked as recommended.

**Background:** Attention has turned in recent years to the broader inclusion of sociology and psychology in medical curricula. Despite this, there is limited published evidence about how best to assess these subjects. This lack of evidence is significant given that most medical schools are likely to include some form of assessment of sociology and psychology, and that sociology and psychology are included in areas examined in admissions tests and in licensing exams.

**Methods:** We ran three one day workshops in the UK (London, Edinburgh and Manchester, June - July 2019), to consult with educators involved in sociology and psychology teaching in medicine on: what methods are being used to assess sociology and psychology in UK undergraduate medical education, and the challenges and opportunities experienced. 36 participants attended the workshops, representing 19 of the 33 UK medical schools. Following the workshops, we collated the notes and presentations in order to develop a summary of current assessment practices and synthesis of the main themes identified.

**Results:** There were many examples of good practice and development of innovative assessments, particularly in the early years of the programmes. At the same time, participants raised several challenges and tensions in relation tothe method, timing, and placement of sociology and psychology assessment. Participants reported that many of these issues related to dominant assessment cultures in medical education. As a result, assessing sociology and psychology in medicine can seem like fitting a square peg into a round hole. Solutions to these challenges may require wider changes to assessment practices and cultures.

**Conclusion:** The challenges shared by participants are evident; nonetheless, there are important opportunities. Our participants were unanimous in their desire to become involved in dialogue and consultation about assessment. This article, reporting on the views of UK SBS educators, is a positive step towards creating a more robust evidence base upon which to engage in these conversations and inform best practice in sociology and psychology assessment.

## Introduction

Attention has turned in recent years to the broader inclusion of sociology and psychology in medical curricula ( Harden and Carr, 2017; Kendall *et al.*, 2020; Robiner, Hong and Ward, 2020). Indeed, it is widely recognized, including by the UK regulatory body, the General Medical Council, that developing students’ competencies in understanding the social and psychological basis of health and medicine, is essential to their future medical practice ( GMC, 2018). Despite this acknowledgement of the place of sociology and psychology in the curriculum, there is limited published evidence about how best to assess these subjects within the context of a medical programme. This lack of evidence is significant given that most medical schools are likely to include some form of assessment of sociology and psychology, given their inclusion in the curriculum. Moreover, sociology and psychology are included in areas to be examined in admissions tests, for example the MCAT exam in the USA, and in licensing exams, including the forthcoming UK Medical Licensing Assessment - a new exam that all final year UK students will be required to sit from 2024 ( GMC, 2021).

Elsewhere we have questioned whether there may be a fundamental mismatch between social and behavioural sciences (SBS) disciplines and biomedical approaches to assessment, meaning that assessing sociology and psychology in medical education can seem like fitting a square peg into a round hole ( Harden, Collett and Kendall, 2018; Harden and Kendall, 2019). In this article we report from a series of workshops in the UK organized by BeSST (Behavioural and Social Sciences Teaching in Medicine). The workshops brought together many of those involved in sociology and psychology teaching in UK medical schools in order to develop a report of current assessment practice. Drawing on the discussions at the workshops, we report key issues relating to the assessment of sociology and psychology in medicine: what is being assessed; how, when and where SBS subjects are assessed; who is involved in assessment; and assessment cultures.

## Methods

BeSST was established in 2002 as a community of practice for sociologists, psychologists, clinicians and others engaged in teaching social and behavioural sciences in medical education. We have 270 members representing all UK medical schools and increasingly we are attracting international members including the United States, Germany, Qatar, Bahrain, and Japan - reflecting the rise in interest in social and behavioural sciences in medical education across the globe.

BeSST has developed a robust, collaborative approach to working, leading to the development of national core curricula in psychology ( Cordingly *et al.*, 2010) and sociology ( Collett *et al*., 2016). Drawing on this approach we organised three workshops to consult with members about assessing sociology and psychology. We considered sending a survey to members, however we decided that face-to-face meetings would be a more effective method for gathering detailed information and providing the opportunity for discussion.

### The workshops

During June and July, 2019 we ran three one day workshops in different parts of the UK (London, Edinburgh and Manchester) to facilitate attendance. Invitations were sent to all medical schools in the UK. Identifying the correct person/people, responsible for sociology/anthropology and psychology teaching was challenging. While it was clearly identified for some, in others we relied on our network of contacts to locate relevant people, or resorted to sending the email to the undergraduate director, where no specific person was listed. In total, 36 participants attended the workshops, representing 19 of the 33 UK medical schools. The disciplines of sociology and psychology were fairly evenly represented across the three workshops.

The main aims of the workshops were: to determine what methods are being used to assess sociology and psychology in UK undergraduate medical education, and the challenges and opportunities experienced. In the morning, participants from each medical school presented an outline of current practice, highlights and challenges. In the afternoon session, we discussed the issues raised by participants in their presentations, identifying both the core challenges and possible opportunities.

At each session, a facilitator took notes from the presentations, questions and discussion. Following the workshops, we collated the notes and presentations in order to develop a summary of current assessment practices and synthesise the main themes identified. To preserve confidentiality, we do not include the names of participants or their affiliation in the presentation of results.

## Results

We present the main themes that arose during the discussions at workshops: what is being assessed; what methods of assessments are used, when and where sociology and psychology are assessed; who is involved in assessment; and assessment cultures. In this section we only present the points raised in the workshops. In the discussion section, we offer a wider commentary on the key issues.

### What is being assessed?

In the workshops we did not focus on what sociology and psychology topics should be assessed because we have previously addressed this in detail when developing the core curricula. Rather, we discussed at a more conceptual level the question of ‘what’ we are seeking to assess when developing our assessment methods for these areas of the curriculum. Specifically, participants discussed the question of whether we are or should be assessing the areas of knowledge, competencies and skills.

There was debate around the question of whether sociology and psychology involve learning and assessing factual knowledge. While participants stressed that sociology and psychology are disciplines with their own knowledge domains and bodies of thought, a knowledge-based approach to learning and assessment was frequently contrasted with what these disciplines can offer medical students. Sociology in particular was described more as a way of thinking; bringing critical insight and relevant theory and concepts to understanding complex issues relating to health, illness and medical practice. It was also noted that as a result, sociology encourages students to adopt a reflexive stance towards their future role as doctors. Some discussed the idea that sociology and psychology are similar to more ‘liberal arts’ disciplines in that they inherently involve personal development. These contentions reflected the belief that it was not simply ‘knowledge’ that was being taught or assessed.

In the workshops participants discussed the significance of competency-based education and the implications for sociology and psychology assessment. It was noted that we need to acknowledge the difference between what students know and understand, and *how* they use it. For example, students may be aware of socioeconomic health inequalities and understand the drivers behind these, but we need also to ensure that they are competent in applying this understanding in practice. Arising from this, one participant noted that we need a form of assessment that can test ‘how’ something is done, or how knowledge is applied in context. Similarly, another participant argued that linking sociology and psychology to clinical reasoning competencies is a useful way to embed the disciplines in competency based teaching and assessment.

Participants also discussed the potential for sociology and psychology to enhance students’ skills, specifically critical thinking, reflection, and communication. For example, it was noted that learning about LGBT+ health can improve students’ communication with LGBT+ patients. At the same time, there was wariness that these areas of the curriculum are not seen only as the place where we teach and assess academic and personal skills.

### What methods of assessment are used?

In Table 1 we present a summary of the modes and timing of assessment reported by workshop participants. Some programmes are new, and so assessment plans for later years were indicative at this stage. The length of the programmes varied: while most were 5 years, some were 6 years including a pre-entry year for widening participation students or because there was an intercalated year for all students; and others offered shorter graduate entry programmes. To simplify reporting, we have collated assessment practices in years 0-2, and years 3-5/6.

**Table 1:** Summary of type and frequency of assessment methods

Year	Assessment Method	Number of Institutions
1-2(3)	MCQs Essay/report Reflective report Short answer questions Project Portfolio OSCE Presentation Case report Key feature problems Patient leaflet	14 6 4 4 2 2 2 1 1 1 1
3-5(6)	MCQs Case report Reflective report Short answer questions Project OSCE Presentation	7 2 1 1 1 1 1

Knowledge tests using various forms of multiple choice questions (MCQs), was the most common method for assessing sociology and psychology, reported by participants. However, the methods used changed between the early and later years of the curriculum. In the early years there was a wider range of other methods used, including reflective essays, short notes questions and patient interview reports. In the later years there was a much stronger reliance on knowledge tests, case reports and some use of clinical examinations, most commonly OSCEs, with far fewer examples of other assessment methods being used. Many of the schools offer sociology and psychology student selected component (SSC) options, whether as a research project or an optional taught course. Most often these take place in years 2 or 3 and are by their nature taken by a small, self-selecting student body.

Despite the emphasis on knowledge tests, there was an overwhelming critical response to this approach. MCQs were thought to focus mainly on simple factual recall rather than on complexity and context which are considered to lie at the heart of sociology and psychology. As participants emphasised, there are rarely right or wrong answers within these disciplines. For such reasons, they reported that it was very difficult to write meaningful SBS MCQs. It was also pointed out that the sociology and psychology GMC graduate outcomes ( GMC, 2018) refer to ‘describe’, ‘explain’, ‘review’ and ‘apply’; outcomes which are difficult to assess using a knowledge test. There was some discussion about whether it was possible to train people to write better sociology and psychology questions, that is, whether the problem lay not in the assessment tool itself, but in how it was being used. However, there was agreement that while there is certainly always scope to improve question writing, MCQs are inherently limited for the reasons given. Moreover, some participants expressed concerns about the implications of using MCQs to assess sociology and psychology; specifically that knowledge-based assessment would drive the students’ learning towards superficial, rote learning of key facts, rather than facilitating them to achieve the intended outcomes.

Despite these apprehensions, it was acknowledged that as more medical schools are focusing on this type of assessment, and because a significant component of the forthcoming UK Medical Licensing Assessment will be comprised of MCQs, there is pressure to produce them. Participants raised specific concerns about whether sufficient (number and range) and appropriate (assessing outcomes) sociology and psychology questions will be included in the MLA. At the moment, there are a limited number of SBS questions that have been approved through the item review groups and many of these are the type of factual recall questions that participants considered to be limited in assessing sociology and psychology. At the same time, there were concerns that if there is not adequate SBS presence in the MLA this may lead to reduced space in the curriculum and less student attention given to these subjects as a result of curricula being focused more on the MLA.

In contrast to the views towards knowledge tests, participants viewed essays, reflective reports and other more discursive assessments as well suited to assessing sociology and psychology. It was noted that such assessment methods offered students the opportunity to draw from relevant conceptual and empirical work to discuss topics in some depth, and to apply their understanding to specific examples. The value of integrating patients’ lived experience into an essay or report was highlighted, as it could help students to appreciate real world complexity. Nonetheless, participants also noted that assessments relating to patients’ stories or patient cases can remain fairly superficial and descriptive, if not linked in some way to the evidence and concepts offered by sociology and psychology. There was some discussion of the potential for using creative methods such as arts or poetry, but there was also concern about a negative response (from students and educators) to the use of such methods of assessment because they are further from a traditional medical education assessment paradigm.

A small number (around ¼) of schools reported that sociology and psychology were reflected in clinical exams. There was recognition of the importance of the clinical encounter as a context in which to assess the application of sociology and psychology; and a desire expressed by participants to see a greater inclusion of SBS in clinical exams. However, there were some concerns expressed about the application of sociology and psychology knowledge and understanding within OSCEs becoming superficial. Similarly, there were concerns that OSCEs may focus too much on assessing some competencies well, such as communication skills, that may be more straightforward to assess in this context, and less on others, particularly the application of an understanding of the influence of social structures (structural competence).

### When and where are sociology and psychology assessed?

Although most programmes did not have a clear pre-clinical/clinical structure, there was evidence of a differential pattern of assessment between the early and later years of the programmes in terms of the methods of assessment, as we have seen, but also with regards to the scope of assessment. All schools included explicit sociology and/or psychology assessment in the early years of the programme but the extent to which the disciplines were present in assessment decreased significantly in the later years; some reported no inclusion in assessment and many said there was a minimal presence. Participants reported feeling less connected to students’ learning in clinical years and not necessarily being fully aware of what is taught. There was a reported sense of the need to continually look for opportunities to find a way into teaching and so assessment.

In part, this idea of finding ‘a way in’, connected to the issue of integrated assessment that was raised as an issue in all the workshops - where sociology and psychology were integrated into, or formed a part of a wider assessment, for example as one domain within an OSCE. Participants noted the opportunity that integrated assessment offered as a way to enhance the perceived relevance, salience and identifiable ‘value’ to students. At the same time, there were also challenges identified. Developing effective integrated assessment was reported as being dependent, to some extent, on the degree of integration of the subjects/topics in the curriculum. The minimal assessment presence in the later, more clinically oriented years of the curriculum reflected the wider challenges of meaningfully embedding sociology and psychology within an integrated curriculum. Participants also noted that developing integrated assessments was complex because it involved a wide range of topics/disciplines, multiple members of staff, often with different expertise, viewpoints and priorities.

### Who should be involved in assessment?

Participants emphasised the importance of assessors being experts in their subject. In part, they emphasized the important role of disciplinary expertise, but they also noted the benefits of working in partnership with clinicians to develop assessments, for example in writing OSCEs. Nevertheless, participants identified challenges arising during such partnerships. In particular, there were some reported tensions between what disciplinary specialists and clinicians considered to be key learning points to be assessed and a risk of clinicians diluting expectations around the assessment of sociology and psychology understanding. Engaging in training with clinicians to communicate expectations and the rationale for assessment criteria was reported to minimize this risk.

Participants also reported resource constraints; many worked in very small teams or were the only sociology or psychology member of medical education faculty. This made the need to draw in others, clinical partners for example, essential for most. Resource constraints also shaped the choice of methods of assessment that could be used; assessment which has low resource requirements such as MCQs, was sometimes considered more feasible than those which are resource intensive, such as essays.

### Assessment culture

The importance of assessment culture in shaping all of the above issues was a core issue discussed in all the workshops. There were several aspects that presented challenges for assessing sociology and psychology that participants raised.

Participants reported a sense of having to compete for space in assessment blueprints and some negativity toward sociology and psychology within the context of that competition; reflected in such reported comments as ‘you can’t assess everything’, and ‘we are training doctors you know’. While all schools had to meet GMC requirements, the space given (or not) to the disciplines was seen as dependent on the views of core people within the programme leadership and those with responsibility for modules or areas of the curriculum. This meant that some participants felt they had limited control over assessment. Many of the programmes did not have a specific external examiner for sociology and psychology, so potential issues that arise may not be subject to external scrutiny.

Medical programmes have lots of moving parts and participants noted that there is often a real inertia in attempts to bring about change. Often particular lectures, topics, and assessments were said to be done because ‘that’s what has always been done', rather than because there was a strong pedagogical rationale. Similarly, participants noted that traditional, positivist trends in medical education assessment and quality assurance processes privileged reductionist thinking and an emphasis on reliability measures, leading to an over reliance on certain methods of assessment, even if, as discussed above, they may not be the most appropriate. Such positivist beliefs were also reported as arising from students when concerns were expressed

The views of students and how these reflected and reinforced the assessment culture was also discussed. Participants noted the often strategic, assessment-driven approach to learning adopted by many students, as reflecting the competitive assessment culture. In contexts where the disciplines are integrated as a minor part of an assessment, students were reported as performing tactically and choosing to ignore or neglect the sociology and psychology elements. There were concerns that because assessment presence and weight are often considered to be a measure of the value of a subject, the minimal presence of sociology and psychology leads students to consider them as being of minimal importance. However, participants also discussed student concerns where sociology and psychology were assessed separately, such that that they had to focus on them or risk failure; this practice was regarded as ‘unfair’ by students because of the subjectivity of marking of non- MCQ assessments, again reflecting the dominant positive paradigm.

## Discussion

Arguably assessment is one of the most dominant and contentious topics within medical education with ongoing debates over the most appropriate methods for determining if students have met learning outcomes or competencies. Yet, there is very limited published evidence about how best to assess SBS subjects. To address this evidence gap, BeSST organized three workshops with SBS educators in the UK to determine what methods are being used to assess sociology and psychology in UK undergraduate medical education and the challenges and opportunities experienced by those involved in developing and delivering assessment. There were many examples of good practice and development of innovative assessments, particularly in the early years of the programmes. At the same time, participants raised several challenges and tensions in relation to the method, timing and placement of sociology and psychology assessment. Participants reported that many of these issues related to dominant assessment cultures.

In the United Kingdom, the national standard for medical education, *Promoting Excellence* ( GMC, 2015), does not specify modes of assessment but instead notes that assessments must be “fair, reliable and valid” ( GMC, 2015, p. 35). van der Vleuten and Schuwirth (2005) state that assessment should demonstrate validity, reliability, feasibility, fairness and acceptability. In UK medical education, the dominant assessment paradigm, reflected in the use of psychometrics to evaluate assessment tools, reflects and reinforces a particular form of learning, ways of knowing and methods of assessment. Underlying this approach is a positivistic belief that there is a single, constant reality or truth which can be generalised, measured and known. In contrast, sociologists and many psychologists tend to adopt a social constructionist perspective which emphasises the context-specific and culture-bound nature of phenomena. These are quite fundamentally different positions which have implications for assessment; each one lends itself to different types of assessments. What is most significant is that, despite some claims of a shift to a ‘post-psychometric era’ ( Hodges, 2013) the participants’ felt that in the UK the dominant mode of assessment is still based on a positivist paradigm. As a result, assessing sociology and psychology in medical education can seem like fitting a square peg into a round hole.

Should we work more towards fitting sociology and psychology outcomes or competencies into this assessment paradigm, that is, should we change the shape of the SBS peg to fit the hole? In some ways when we write MCQ questions for knowledge tests that is exactly what we are doing. On the other hand, as we have seen, this is problematic because many areas of sociology and psychology learning are thought to be unsuited to assessment via knowledge tests. For example, while it may be possible to devise some MCQs that relate to the social patterning of illness among different ethnic groups, it is not possible to assess students’ understanding of the meanings, drivers, and experiences of racism that may underpin these patterns via an MCQ. In order to assess competencies that relate to key learning points such as this, now often referred to as structural competency ( Metzl and Hansen, 2014), we need to change the hole, not the peg. This is particularly concerning in the UK in the context of the new MLA exam of which MCQs will form a significant part.

We noted that participants saw potential for sociology and psychology assessment via clinical assessments, specifically OSCEs. The appeal of this method of assessment was in part that it allowed for the assessment of the application of knowledge, but also that it was an opportunity to embed sociology and psychology in clinical, and often later years, assessments. However, there are challenges to this. Epstein (2007) contend that assessment of competencies should assess actual performance. In OSCEs, what is expected from a students’ performance has to be categorized and codified in such a way as to make assessment fair and reliable. For this reason, the application of sociology and psychology knowledge that often involves an understanding of complexity of social contexts or structural constraints, may be distilled to a single criterion; for example, that the student should ask about the patient's family background or occupation. While appropriate in many contexts, this would often result in the assessment of SBS at a superficial level. Therefore, while there may be opportunities for the SBS peg and the OSCE hole to both modify somewhat and fit nicely together, this may not be case for all sociology and psychology outcomes.

This leads us to question whether it is the hole rather than the peg that should change? At a basic level this may involve the development of assessment methods that allow us to appropriately assess sociology and psychology competencies. Kuper *et al.* (2007) argue that we should consider alternative paradigms of assessment (not just in relation to SBS) that may better reflect the context and complexities of clinical work in general. The perceived need to standardise and control assessments to ensure reliability may not fit well with the need to be able to ‘test’ students’ competencies in real and changing clinical situations. Indeed, Kuper *et al.* (2007) argue that competencies should be seen as socially constructed rather than simply a measure of an individual's ability. For example, empathy is demonstrated in the interaction with a patient and so is constructed through the perceptions that the doctor and patient have of one another, and of the situation, as well as what empathy means to each of them. One suggestion they offer to address this, is that we draw on qualitative research methodologies, including participant observation and interviewing, to develop modes of assessing in situ, such as on the ward. This suggested change to the assessment hole would potentially fit well with the contextual, structural and relational SBS peg. On the other hand, given the challenges participants described relating to assessment cultures - the relatively low place of sociology and psychology in the curricula hierarchies and the limited weight that small SBS teams can bring to advocate for their disciplines - there would be many barriers to the introduction of assessments of the kind that Kuper *et al.* (2007) suggest.

The Consensus Framework for Good Assessment ( Norcini *et al.,* 2011; 2018) provides clear guidance on what can be considered best practice at the level of individual assessment and systems of assessments. However, there is no consideration of SBS or the specific issues discussed in the workshops reported here. This leaves many areas worthy of further investigation including: do SBS educators agree with the view that the catalytic effect of education is less significant in summative assessment? What is the place of SBS and SBS educators as stakeholders in systems of assessment? Is the assessment process and results credible and evidence based in relation to SBS? Do SBS educators experience the system of assessment as transparent and free from bias? Building the evidence relating to sociology and psychology assessment may perhaps feed into future iterations of such consensus frameworks.

## Conclusion

The workshops brought together dedicated SBS experts working in medical schools across the UK and highlighted the commitment, enthusiasm and innovation of those working in these roles. The challenges shared by all are evident. Nonetheless, there are important opportunities. Our participants were unanimous in their desire to become involved in dialogue and consultation about SBS assessment, not only within their own schools, but also with governing bodies such as the GMC and the Medical Schools Council, as well as with colleagues in other UK medical schools and across the globe. This article, reporting on the views of UK SBS educators, is a positive step towards creating a more robust evidence base upon which to engage in these conversations and inform best practice in sociology and psychology assessment.

## Take Home Messages

• MCQs remain the most frequently used method for assessing sociology and psychology despite the views of disciplinary experts that this is not an appropriate method to assess these subjects.
• Sociology and psychology have minimal presence in clinical assessments, and in later years’ assessments.
• Assessment cultures influence the space given to sociology and psychology assessment, the response of staff and students to alternative assessment methods, and the opportunities for change, making it feel like fitting a square peg into a round hole.
• There is a need for a more robust evidence base to inform best practice in sociology and psychology assessment.

## Notes On Contributors

**Jeni Harden** is Senior Lecturer in Social Sciences and Health, University of Edinburgh, Medical School and is co-chair of Behavioural and Social Sciences Teaching (BeSST) in Medicine. ORCID ID: https://orcid.org/0000-0002-6011-6254

**Tracey Collett** is Associate Professor of the Sociology of Health and Illness at the University of Plymouth and is co-chair of Behavioural and Social Sciences Teaching (BeSST) in Medicine. ORCID ID: https://orcid.org/0000-0002-8541-0417

**Simon Forrest** is Professor of Sociology, Principal of College of St Hild and St Bede at Durham University, and is co-chair of Behavioural and Social Sciences Teaching (BeSST) in Medicine. ORCID ID: https://orcid.org/0000-0002-3040-479X

**Kathleen Kendall** is Associate Professor of Sociology as Applied to Medicine in the Faculty of Medicine at the University of Southampton and is co-chair of Behavioural and Social Sciences Teaching (BeSST) in Medicine. ORCID ID: https://orcid.org/0000-0002-4235-0741
