# Supplementary material for: Assessing sociology and psychology in UK undergraduate medical education: Square peg in a round hole?
Source: MedEdPublish (2016). 2021 May 17;10:133. [Version 1] doi: 10.15694/mep.2021.000133.1 (PMC10939541; doi:10.15694/mep.2021.000133.1)
Supplement: Peer review history

# Reviewer report: Reviewer response for version 1

**DOI:** 10.21956/mep.19102.r27540

**Published:** 2021-07-14

**Authors:** P Ravi Shankar

**Affiliations:** 1 American International Medical University

This review has been migrated. The reviewer awarded 4 stars out of 5 This is an interesting article about the assessment of Sociology and Psychology subjects in UK medical schools. The article is well-written and presented. The introduction of a licensing exam at the end of the course which may be predominantly using MCQs may promote strategic learning among students as the authors have mentioned. My experience in offshore Caribbean medical schools highlights the huge importance given to Step 1 of the USMLE and how it influences the importance and time students give to different subjects and concepts. One of the advantages of an MCQ from the perspective of a developer of a high-stakes licensing exam is defensibility. If well written and vetted an MCQ will only have one correct answer. Despite the use of rubrics, marking a project or an essay assignment is still more subjective. I also agree with the authors about the relatively less importance accorded to the social and behavioral sciences in the medical curriculum. Also, in an increasingly crowded curriculum, each discipline/subject will have to fight for space and recognition. The knowledge explosion makes it more likely that the curriculum will be even more packed in the future. The article will be of general interest to medical educators.

Reviewer Expertise:

NA

No decision status is available

# Reviewer report: Reviewer response for version 1

**DOI:** 10.21956/mep.19102.r27539

**Published:** 2021-07-13

**Authors:** Richard Hays

**Affiliations:** 1 James Cook University

This review has been migrated. The reviewer awarded 4 stars out of 5 In my curriculum designer role I have always included behavioural and social sciences as core material that must be delivered and assessed. My strategy was a high degree of curriculum integration, such that students encountered the relevant behavioural and social sciences in a context that reflected future practice. A consistent message from to students has been 'my success as a doctor relies not just on my understanding of the biomedical sciences but also my ability to understand and work with people as they move through the complexities of the health care system. Behavioural and social sciences are the building bocks of that understanding. Not everyone agrees with this approach, as teaching this way requires a different approach to that in behavioural science programs but my experience with those more traditional teaching methods has not been good. This research comes from those who know best about teaching and assessing behavioural and social sciences in medical programs. The positive note is that more of it happens than many think, the negative note is relatively poor recognition by faculty and students of its importance. I recommend this article to all those engaged in curriculum design for medical programs.

Reviewer Expertise:

NA

No decision status is available
